# Emerging Strategies to Combat β-Lactamase Producing ESKAPE Pathogens

**DOI:** 10.3390/ijms21228527

**Published:** 2020-11-12

**Authors:** Corneliu Ovidiu Vrancianu, Irina Gheorghe, Elena-Georgiana Dobre, Ilda Czobor Barbu, Roxana Elena Cristian, Marcela Popa, Sang Hee Lee, Carmen Limban, Ilinca Margareta Vlad, Mariana Carmen Chifiriuc

**Affiliations:** 1 Microbiology Immunology Department and The Research Institute of the University of Bucharest, Faculty of Biology, University of Bucharest, 020956 Bucharest, Romania; ovidiu.vrancianu@yahoo.com (C.O.V.); dobregeorgiana_95@yahoo.com (E.-G.D.); ilda.czobor@yahoo.com (I.C.B.); bmarcelica@yahoo.com (M.P.); carmen.chifiriuc@bio.unibuc.ro (M.C.C.); 2 Department of Biochemistry and Molecular Biology, Faculty of Biology, University of Bucharest, 020956 Bucharest, Romania; roxana.cristian95@yahoo.com; 3 Department of Biological Sciences, Myongji University, 03674 Myongjiro, Yongin 449-728, Gyeonggido, Korea; sangheelee@mju.ac.kr; 4 National Leading Research Laboratory, Department of Biological Sciences, Myongji University, 116 Myongjiro, Yongin 17058, Gyeonggido, Korea; 5 Department of Pharmaceutical Chemistry, Faculty of Pharmacy, “Carol Davila” University of Medicine and Pharmacy, Traian Vuia no.6, 020956 Bucharest, Romania; carmen_limban@yahoo.com (C.L.); ilincamargaretavlad@gmail.com (I.M.V.); 6 Academy of Romanian Scientists, 030167 Bucharest, Romania

**Keywords:** ESKAPE, inhibitors, β-lactamase, antimicrobial resistance, vaccination

## Abstract

Since the discovery of penicillin by Alexander Fleming in 1929 as a therapeutic agent against staphylococci, β-lactam antibiotics (BLAs) remained the most successful antibiotic classes against the majority of bacterial strains, reaching a percentage of 65% of all medical prescriptions. Unfortunately, the emergence and diversification of β-lactamases pose indefinite health issues, limiting the clinical effectiveness of all current BLAs. One solution is to develop β-lactamase inhibitors (BLIs) capable of restoring the activity of β-lactam drugs. In this review, we will briefly present the older and new BLAs classes, their mechanisms of action, and an update of the BLIs capable of restoring the activity of β-lactam drugs against ESKAPE (*Enterococcus* spp., *Staphylococcus aureus, Klebsiella pneumoniae, Acinetobacter baumannii, Pseudomonas aeruginosa*, and *Enterobacter* spp.) pathogens. Subsequently, we will discuss several promising alternative approaches such as bacteriophages, antimicrobial peptides, nanoparticles, CRISPR (clustered regularly interspaced short palindromic repeats) cas technology, or vaccination developed to limit antimicrobial resistance in this endless fight against Gram-negative pathogens.

## 1. Introduction

Since the discovery of penicillin by Alexander Fleming in 1929 as a therapeutic agent against staphylococci, β-lactam antibiotics (BLAs) remained the most successful antibiotic classes. BLAs are the most widely used antibacterial agents against infectious diseases, reaching a percentage of 65% of all medical prescriptions. In general, they are well tolerated and have high efficiency in eliminating resistant bacteria. However, side effects such as allergic responses or delayed hypersensitivity reactions could often occur [1]. BLA’s mechanism is based on blocking the formation of the bacterial cell wall following covalent binding to penicillin-binding proteins (PBPs), enzymes involved in the final stages of cross-linking of the peptidoglycan layer (PG) in the bacterial cell wall, both of Gram-negative and Gram-positive bacteria.

Initially, the inhibition mechanism of PG transpeptidation by penicillin was described in 1965 by Tipper and Strominger [2]. They observed a structural similarity of penicillin G to the D-ALA-D-ALA dipeptide from the PG structure. This mechanism involves either binding penicillin to an active site of serine located in functional PBPs or binding to an allosteric site of PBP2a from *Staphyloccocus aureus*. In the first case, the penicillin-binding to the active site determines the enzyme’s acylation and the antibiotic hydrolysis [3]. In the second case, binding to the allosteric site leads to an increased sensitivity response of the body [4,5]. Inactivation of PBPs by BLAs causes the accumulation of PG precursors leading to the hydrolases activation in the cell wall, which also degrade the intact PG, causing the lysis of the actively dividing cells [6]. In Gram-positive bacteria, the PG is 50–100 times thicker than in Gram-negative and strongly intertwined, which maintains structural integrity in Gram-positive [7]. Therefore, BLAs have a more decisive action on Gram-positive bacteria. It is also worth mentioning that all the Gram-negative pathogens present an additional membrane layer often referred to as the “outer membrane” [8]. This asymmetrical lipid bilayer composed mainly of glycolipid lipopolysaccharides (LPS) and glycerol phospholipids acts as a robust barrier for protection against various environmental stimuli and toxic compounds, including antibiotics, whose targets are particularly located beyond this layer [9]. The barrier function of the outer membrane is responsible for the endotoxin shock associated with the septicaemia caused by Gram-negative organisms and proteins that mediate the passive or active uptake of small molecules [10].

BLAs have saved countless lives by now and remain the backbone of therapy for the majority of bacterial infections, including those caused by ESKAPE pathogens. The Gram-negative group, that encompasses the Gram-negative ESKAPE pathogens withstand resistance to a broad group of antimicrobial compounds, including carbapenems, which are considered “last resort” BLAs [11]. The phenomenon of antimicrobial resistance is a multifaceted one and multiple mechanisms have been associated with BLAs failure, including the production of β-lactamases (enzymes able to hydrolyze the BLAs), structural alterations in PBPs, decreased expression of outer membrane porins (OMPs), and increased drug efflux. Among all of them, β-lactamase-mediated resistance to BLAs is by far the most common and important mechanism of resistance in Gram-negative species [12]. In Gram-negative bugs, the enzymatic resistance may be mediated by either plasmid- or chromosomal β-lactamases; notably, inducible expression of chromosomal β-lactamases is common in almost all Gram-negative microbes, while plasmid-mediated enzymes are usually expressed constitutively [13]. The plasmidial enzymes are usually class A enzymes, whereas the chromosomal β-lactamases belong to class C enzymes [14]. The epidemiological dimension of increased resistance to BLAs is mainly linked with the global spread of plasmid-mediated β-lactamases, such as the CTX-M-type enzymes [15,16]. Unfortunately, the emergence and diversification of β-lactamases threaten the clinical effectiveness of all current BLAs, and one solution is to develop β-lactamase inhibitors (BLIs) capable of restoring the activity of β-lactam drugs or alternatively to develop new representatives from this class. In this review, we will briefly present the older and new BLAs classes, their mechanisms of action, and an update of the BLIs capable of restoring the activity of β-lactam drugs against ESKAPE pathogens. Subsequently, we will discuss several other promising alternative approaches such as bacteriophages, antimicrobial peptides, nanoparticles, CRISPR cas technology, or vaccination developed to limit antimicrobial resistance in this endless fight against these pathogens.

## 2. Classification of β-Lactam Antibiotics (BLA)

Depending on the molecular weight, PBPs are divided into two classes: low molecular weight PBPs, which generally function as carboxypeptidases, and high molecular weight PBPs divided into two classes, A and B [17]. Class A includes bifunctional enzymes, consisting of a transpeptidase domain and a transglycosylase domain. Class B comprises transpeptidases containing the dipeptide D-Ala-D-Ala. A unique set of PBPs for each bacterial species can contain up to eight enzymes per species [18]. Examples of these PBSs in Gram-negative bacteria are PBP1a, PBP1b, PBP2, and PBP3. Their inhibition blocks the cellular division, causing shape changes (e.g., the occurrence of filamentous forms following the β-lactams treatment) or bacterial cell lysis.

### 2.1. Penicillins

**Figure 1:**
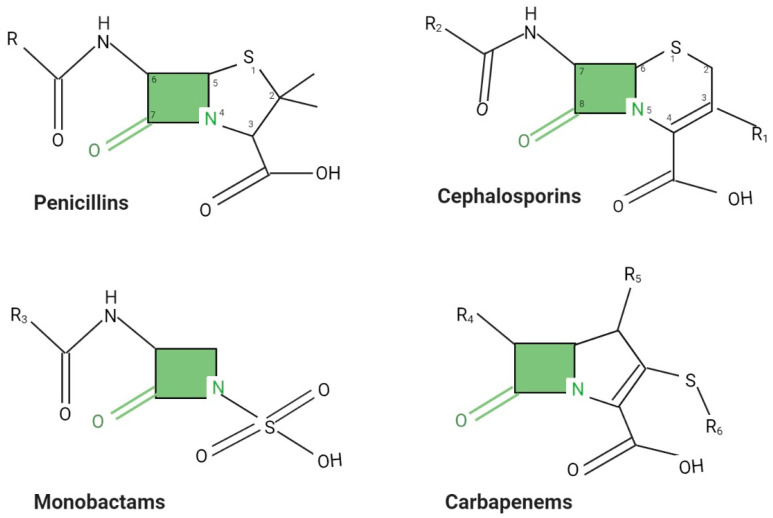
The chemical structure of the main classes of BLAs. The β-lactam ring is stained green for all these representatives.

Either natural or semi-synthetic, penicillins are the longest-used antibiotics in managing bacterial infections globally, being suitable even in the pediatric context [1]. Penicillins are part of the penam group and contain a β-lactam ring, a thiazoldine core, and a side chain with variable dimensions that differentiates penicillins from each other [19]. The side chain is responsible for the biological activities and chemical properties of different penicillins (Figure 1) [20]. Penicillins are classified as natural (penicillin G and penicillin V) or semi-synthetic, including penicillinase-resistant-penicillins, aminopenicillins, and antipseudomonal penicillins.

Natural penicillins such as benzylpenicillin (penicillin G) and phenoxymethylpenicillin (penicillin V) have low oral bioavailability, and therefore, are usually administered intravenously or intramuscularly. However, they are useful only in treating Gram-positive cocci and streptococci and several other non-penicillinase-producing microorganisms [1]. After prolonged exposure to natural penicillins, many penicillinase-producing strains have also emerged among Gram-positive rods. This problem has fueled the search for new semi-synthetic derivatives resistant to β-lactamases, thus giving rise to the second generation of penicillins including oxacillin, dicloxacillin, and methicillin [20]. Although more stable, these drugs were less effective than initially anticipated. They brought a slight improvement in managing penicillinase-susceptible Gram-positive microorganisms compared to natural penicillins and no activity against Gram-negative species.

Furthermore, many studies have reported methicillin-resistant *S. aureus* (MRSA) strains occurring throughout the world. MRSA can cause life-threatening infections in hospitalized and non-hospitalized patients, which, in turn, has limited methicillin use in this clinical setting [21]. Methicillin resistance is correlated with the abundant production of an altered PBP protein: PBP2a, which can replace other PBPs and confer resistance to all BLAs [22].

The introduction of the third generation of penicillins, aminopenicillins (ampicillin and amoxicillin), has brought considerable advantages over its predecessors. Aminopenicillins showed increased activity against *Enterococcus* spp. and several Gram-negative species such as *Haemophilus influenzae, Escherichia coli, Salmonella* spp., and *Shigella* spp. [20,23]. Ampicillin is usually given parenterally, whereas amoxicillin is orally administered. However, their stability is relatively weak, being susceptible to the attack of staphylococcal penicillinase and β-lactamases produced by Gram-negative bacteria [6]. The limited efficiency of penicillins against Gram-negative organisms has considerably accelerated the pharmacological research in the field, leading to new classes of compounds with an enhanced spectrum of action. Such examples are the antipseudomonal penicillins carboxypenicillins (ticarcillin and carbenicillin) and the ureidopenicillin piperacillin [23].

Interestingly, in recent years, it has been observed that the effectiveness of penicillin-based regimens can be accelerated by combining them with β-lactamase inhibitors (BLIs), such as clavulanic acid, tazobactam, and sulbactam. BLIs act mainly on enzymes, allowing BLAs to exert their antibacterial effects [12]. Piperacillin is used in conjunction with tazobactam in the management of appendicitis, skin, and soft tissue infections, as well as community and hospital-acquired pneumonia (CAP and HAP) [12,24]. Ampicillin-sulbactam combinations administrated both intravenously and intramuscularly effectively treat gynecological, intra-abdominal, and dermatological infections [12]. Clavulanate can be administered orally in conjunction with amoxicillin (Augmentin) or parenterally, combined with ticarcillin; in these formulations, it can be used to treat from uncomplicated sinusitis and otitis to complicated sepsis [12,25].

### 2.2. Cephalosporins

Cephalosporins are another category of BLAs isolated from *Acremonium chrysogenum*, also known as *Cephalosporium* spp. There are six generations of cephalosporins, and each generation is administered in a specific clinical context. The basic structure of cephalosporins is the 7-aminocephalosporanic acid (7-ACA). The chemical changes in position 7 of the β-lactam nucleus cause the pharmacological properties of cephalosporins and help their stratification (Figure 1) [26]. The first and second generation’s cephalosporins are potent against Gram-positive rods, while the third and fourth generations are more active against Gram-negative species. The identification of ceftaroline, an effective anti-MRSA cephalosporin that displays an increased affinity for PBP2a, marked the transition to the fifth-generation cephalosporins [27]. Cephalosporins are much more resistant to β-lactamases and have a broader spectrum of action than penicillins; however, extended-spectrum β-lactamases (ESBLs) may interfere with the therapeutic efficacy of even the third-generation cephalosporins [6].

The first-generation cephalosporins include cephalothin, cefazolin, cephalexin, cephapirin, cephradine, and cefadroxil; they have great action on methicillin-susceptible cocci and moderate activity on several enterobacteria (*E. coli, Klebsiella* spp., and *Proteus mirabilis*). These cephalosporins have multiple indications, being recommended in the prophylaxis of post-surgical infections in the clinical management of otitis media, bacteremia, biliary tract infections, and many infections in the cardiac, respiratory, intra-abdominal, orthopedic, dermatological, and genitourinary settings [28]. However, first-generation cephalosporins cannot cross the blood–brain barrier (BBB) and are often associated with recurrent infections [6].

Second-generation cephalosporins are subdivided into two major groups: ‘true’-second-generation cephalosporins and the cephamycins. The subgroup of true cephalosporins includes cefuroxime and cefprozil, whereas the cephamycins are represented by cefmetazole, cefoxitin, cefminox, and cefotetan. Usually, most second-generation compounds have similar indications as to their predecessors. However, the second generation of cephalosporins has a broader spectrum of action on some Gram-negative rods species and on *H. influenzae* and *Neisseria* spp. [23]. A remarkable compound in this group is cefoxitin, active on both Gram-positive and Gram-negative anaerobes. It is also extremely potent in the complications associated with Lyme disease [27,29]. 

The third-generation cephalosporins include cefotaxime, ceftriaxone, ceftazidime, ceftazidime, ceftazidime/avibactam, cefdinir, cefixime, and cefoperazone. They are much more resistant to β-lactamases produced by Gram-negative bacilli but can be hydrolyzed by ESBLs, such as carbapenemases and AmpC enzymes [30]. Remarkably, ceftazidime is one of the most active compounds from this class against *Pseudomonas aeruginosa*; this activity is mainly due to the methoxyamino group’s replacement with a dimethylacetic acid residue [23]. Due to their broad spectrum of action, these cephalosporins are recommended for the treatment of a wide range of infections, including infectious endocarditis, spontaneous bacterial peritonitis, digestive tract infections, urological infections, human and animal bites, genital tract infections, and other sexually transmitted infections [29,31,32,33,34,35]. Notably, due to their ability to reach a sufficient concentration in the central nervous system, they are also recommended to treat meningitis caused by Gram-negative bacilli [23].

The fourth generation of cephalosporins, which includes cefpirome and cefepime, has a broader spectrum of action than their predecessors and remarkable stability to the action of chromosomal or plasmid-mediated β-lactamases [1]. Cefepime is active against an increased number of *Enterobacteriaceae*, *P. aeruginosa*, and various Gram-negative β-lactamases producing strains [27]. Interestingly, due to the remarkable penetration rate through OmpF outer-membrane porin, cefepime has the lowest MIC values against *Enterobacteriaceae* of all broad-spectrum cephalosporins [36,37]. Fourth-generation cephalosporins are also more potent against Gram-positive cocci and are usually used as critical interventional therapy when other cephalosporins cease to function [6]. 

The fifth-generation cephalosporins include representatives such as ceftaroline, ceftobiprole, and ceftolozane. These compounds are highly effective against Gram-positive cocci (e.g., *Streptococcus* spp., methicillin-susceptible *S. aureus*- MSSA, MRSA) and Gram-negative bacilli, except for ESBLs- and AmpC-producing strains such as *Acinetobacter baumannii* [38]. Ceftaroline fosamil is an N-phospho prodrug metabolized in vivo to the active compound, ceftaroline, after intravenous administration. Ceftaroline is a broad-spectrum cephalosporin that has been developed to target resistant bacterial strains, especially MRSA. This agent’s effectiveness is mainly due to the high affinity for all six PBPs, especially PBP2a. In addition to its activity on MRSA, ceftaroline has also been documented to be effective against vancomycin-intermediate *S. aureus* (VISA), vancomycin-resistant *S. aureus* (VRSA), various *Staphylococcus* spp. such as *S. hominis, S. epidermis,* and *S. hemolyticus* and also on *H. influenzae* [1]. Notably, ceftaroline is 2–4 times more effective in inhibiting the microbial growth of staphylococci and streptococci than ceftobiprole and is widely used in the management of CAP and HAP [39].

Ceftobiprole is a metabolite prodrug of the ceftobiprole medocaril, which is also parenterally administered. Its spectrum of activity includes mainly the same species on which ceftaroline acts, with small differences in anaerobic bacteria [23,28]. In addition to the increased affinity for PBP2a in MRSA, ceftobiprole has been shown to bind to PBP2a in *S. epidermidis* and PBP2x in penicillin-resistant *S. pneumoniae* [40,41,42]. Interestingly, ceftobiprole is not hydrolyzed by class A β-lactamases (TEM), AmpC-β-lactamases, and Staphylococcal PC1 enzymes, but remains vulnerable to the action of class B, D of β-lactamases, and ESBLs [43]. Interestingly, ceftobiprole has a lower MIC value than ceftaroline in treating *A. baumanii* or *P. aeruginosa* infections [1,6].

Ceftolozane, administered in conjunction with tazobactam, is a cephalosporin that has not been included in the cephalosporin generation series. This combination is distinguished from all other agents by its activity against various ESBLs-producing enteric species, including *P. aeruginosa* [44]. Ceftolozane/tazobactam was approved by the Food and Drug Administration (FDA) in 2014 to treat abdominal infections, pyelonephritis, and other complicated urinary tract infections (cUTIs) [1]. 

Another compound recently added to the cephalosporin arsenal is cefiderocol. It has a structure similar to that of cefepime and ceftazidime, but which also has a siderophore catechol group, which allows it to penetrate the periplasmic space by exploiting the ion iron transfer system [45]. The FDA recently approved it in September 2019, being one of the strongest β-lactams with remarkable structural stability against various Ambler class A, C, D β-lactamases, and some β-lactamases from class B. This confers activity on multi-drug resistant (MDR) *A. baumannii*, *P. aeruginosa*, and *Stenotrophomonas maltophilia* [46,47]. Cefiderocol is more potent than ceftazidime-avibactam (CAZ-AVI) and meropenem in the treatment of *A. baumannii*, including strains resistant to meropenem or MDR. Also, the antimicrobial activity of cefiderocol is superior to CAZ-AVI in isolates not susceptible to meropenem and *K. pneumoniae* carbapenemase (KPC-) producing *Enterobacteriaceae* [46].

Interestingly, although cefiderocol showed superior efficacy than CAZ-AVI in *P. aeruginosa*, several strains acquired resistance to this compound. The main mechanisms reported were the reduction of the components of the ion transport system, and mutations in these components positioned in the bacterial outer membrane [48,49]. Potential clinical applications of cefiderocol include, but are not limited to, the treatment of HAP, ventilator-associated pneumonia (VAP), and cUTIs with limited or no treatment options.

### 2.3. Monobactams

Monobactams, or monocyclic β-lactams, are active against Gram-negative rods and have virtually no activity on anaerobic and Gram-positive microorganisms (Figure 1) [23]. Aztreonam is one of the archetypal representatives of this group, being the only one currently approved. It is resistant to several types of β-lactamases and is used successfully against Gram-negative bacteria, including *P. aeruginosa* [1]. The antibacterial properties of aztreonam are due to its increased affinity for PBP3 and moderate affinity for PBP1a in Gram-negative bacilli [50]. In routine clinical practice, aztreonam is recommended to manage patients with complicated infections caused by Gram-negative rods which does not tolerate penicillins and cephalosporins [51]. Although aztreonam is resistant to metallo-β-lactamases (MBLs) (Imipenemase-IMP, VIM-Verona Imipenemase, NDM-New Delhi MBL), its efficiency against MDR and extensively drug-resistant (XDR) microorganisms is still questionable since a significant proportion of MBL producers co-produce ESBLs, thus making them aztreonam resistant [52,53].

BAL30072 is a new monocyclic β-lactam belonging to the class of sulfactams. The siderophore group from its structure is essential to forming the complex with iron ions and efficient penetration into the periplasmic space. Besides the increased spectrum of aztreonam action, BAL30072 brings additional activity on non-fermenting Gram-negative bacteria [54]. Notably, available preclinical studies to date potentiate that this compound is potent against several carbapenem-resistant *A. baumannii* (CRAB) clones and MBL-producing *P. aeruginosa* strains [54,55,56]. Additionally, it has been reported that BAL20072 is hydrolyzed almost 3000-times less efficiently by KPC-2 than aztreonam [55].

### 2.4. Carbapenems

Carbapenems, including imipenem, ertapenem, meropenem, and doripenem, are the most potent β-lactams due to their increased resistance to most existing β-lactamases, including ESBLs. They distinguish from other β-lactams by having a carbon atom that replaces the sulfur or oxygen atom at the C-1 of the five-membered penicillin-like ring and a hydroxyethyl group in *trans* configuration at C-6 (Figure 1). Due to the increased penetration power through the outer membrane, formidable stability to the action of β-lactamases, and increased affinity for almost all PBPs, carbapenems are potent against Gram-positive, Gram-negative, aerobic, and anaerobic microorganisms. However, carbapenems are restricted only to complicated infections caused by *E. coli*, *K. pneumoniae*, and *P. aeruginosa*. Surprisingly, carbapenems are ineffective against MRSA, *E. faecium*, and several Gram-negative aerobic rods, such as *Burkholderia cepacia* [23].

Meropenem and ertapenem are very active on Gram-negative microorganisms, while imipenem and doripenem only on Gram-positive bacteria. Ertapenem, imipenem, and meropenem have a higher efficacy against *Enterococcus* spp., *Acinetobacter* spp., and *P. aeruginosa* [57]. However, doripenem remains the most stable carbapenem to the action of β-lactamases [58] and has been documented to have lower MIC values than imipenem and meropenem on two notorious ESKAPE pathogens: *P. aeruginosa* and *A. baumannii* [59,60]. However, several studies report carbapenemases in various Gram-negative species; this is of particular importance. Thus, these bacteria become refractory to almost all available BLAs and other classes of compounds, such as fluoroquinolones and aminoglycosides [61,62].

Around the 2000s, the United States firstly reported a *K. pneumoniae* strain carrying a class A β-lactamase-encoding plasmid, capable of hydrolyzing penicillins, cephalosporins, and carbapenems [63,64]. As more than one-third of *K. pneumoniae* isolates are carbapenemase producers, the spread of these strains pose a global epidemiological challenge [65]. Until now, many other carbapenemases have been identified around the world. For example, two carbapenemase encoding genes *bla*_NDM–1_ and *bla*_IMP–4_ have been documented in *K. pneumoniae* producing strains [66]; additionally, two other carbapenemase genes *bla*_KPC_ and *bla*_NDM_ have been reported in *Enterobacter cloacae* [67]. Further complicating this scenario, an isolate of *K. oxytoca* was shown to produce three types of carbapenemases KPC-2, NDM-1, and IMP-4. Plasmids carrying these three resistance genes have been subsequently reported in other *Enterobacteriaceae* strains [68,69].

In Japan, there have been approved two other carbapenems very similar to meropenem and doripenem, namely biapenem [57] (excellent stability to MBLs) and tebipenem (with deficient antipseudomonal activity) [1,70].

## 3. β-Lactamases in Gram-Negative Bacteria

β-lactamases inhibit the β -lactams antimicrobial activity by dissociating the -CO-NH bond at alanyl-alanine dimer level during the PG synthesis. Due to their steric omology, BLAs bind to the alanyl-alanine dimer in a similar region as PBP. On the other hand, β-lactamases and PBPs have similar structures and have common peptidase activity, leading to the idea that β-lactamases were derived during the evolution of PBP [71] (Figure 1).

**Figure 2:**
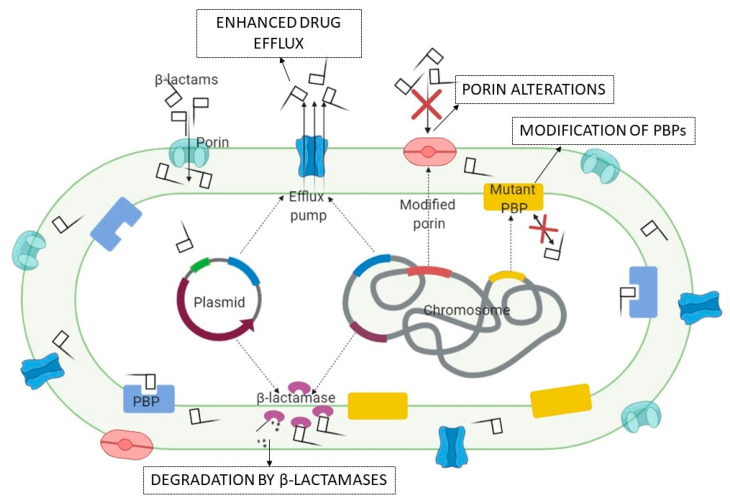
Most common mechanisms of β-lactam resistance in ESKAPE pathogens. Figure created with https://biorender.com/.

In Gram-negative bacteria, β-lactamases have played a significant role over time, representing the main mechanism of resistance to BLAs (Figure 2). The first β-lactamase reported was discovered by Abraham and Chain in 1940 in *Bacillus coli* [72], today considered class C cephalosporinase from *E. coli*. In general, enzymatic resistance to BLAs has been associated with Gram-negative pathogens, many species, such as *P. aeruginosa* and several enteric bacteria having been shown to produce chromosomal inducible β-lactamases [73]. However, one acute problem in the case of β-lactamases is represented by the enzymes encoded by genes located on mobile genetic elements (MGE) that could be transferred by horizontal gene transfer (HGT). In the early 1980s, the transfer of β-lactamases was observed in only a few enterococcal strains [74]. Subsequently, the spread of β -lactamases through MGE proved to be the most important resistance mechanism in Gram-negative bacteria.

### 3.1. Origins

β-lactamases are enzymes with a diverse molecular structure whose common feature is their ability to degrade the BLAs’ structure. Although in 1979 [75], it was specified that β-lactamases appeared with the discovery of the first enzyme capable of degrading penicillin by Abraham and Chain [72], phylogenetic analyses estimated that β-lactamases date to about 2 billion years [76]. The analysis of some permafrost sediments in Canada dating back about 30,000 years and some sediments in Papua New Guinea, dating back about 10,000 years, led to the discovery of amino acid sequences with high similarity with TEM type [77,78]. Metagenomic analysis of ancient samples led to the detection of MBLs in a bone sample from the 14th century [79]. β-lactamase production has been shown in soil and ice core samples in Antarctica and South America populations that have not been administered at all or very rarely commercial BLAs [80,81], thus proving the existence of β-lactamases even in the absence of the selective pressure exerted by antibiotics used in therapy. Interestingly, in studies that analyzed ice samples, MBLs such as IMP, a β-lactamase less commonly involved in BLAs resistance in clinical isolates, were discovered [80].

### 3.2. Classification

**Figure 3:**
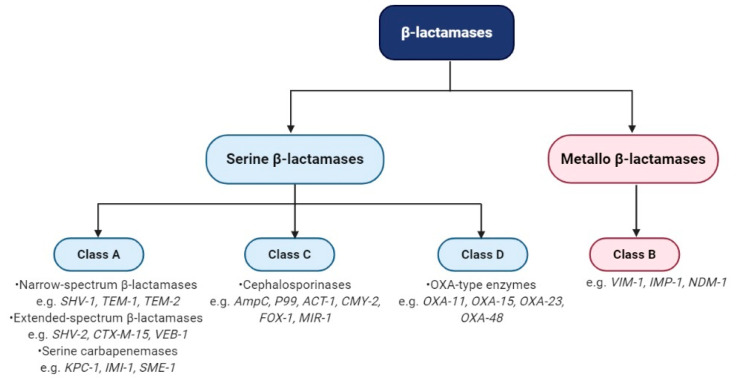
Ambler classification system of β-lactamases.

In general, β-lactamases are classified biochemically into two broad categories, depending on how they perform the hydrolysis of the β-lactam ring. β-lactamases can perform hydrolysis either by forming an acyl-enzyme with an active serine site [82] or by a hydrolysis reaction based on zinc ions from the active sites of MBLs [83]. Initially, Sawai et al. classified β-lactamases into penicillinases and cephalosporinases, depending on the substrate [84]. In 1976, the introduction of isoelectric focusing (IEF) allowing the analysis of the amino acid sequences of key β-lactamases [64]. Ambler made the first molecular classification of β-lactamases in Gram-negative bacteria that divided into four classes, A, B, C, and D. For classes A, C, and D, the active enzyme site contains serine and class B includes Zn-dependent metallo-enzymes (Figure 3) [85]. Between 1979 and 1985, almost 1800 enteric bacteria were analyzed based on IEF profiles to observe the presence of β-lactamases [86,87,88,89,90]. 63% of the analyzed isolates showed *bla*_TEM-1_ and *bla*_TEM-2_ genes, 9.9% of the strains showed SHV-1, and 7.8% showed OXA type enzymes. These studies laid the groundwork for the subsequent complex characterization of β-lactamases. Furthermore, besides the common β-lactamases TEM and SHV, the ESBLs, especially those from the CTX-M family, have been found as essential enzymes responsible for Gram-negative rod resistance [91,92].

Class C-type cephalosporinases have been implicated over time in resistance to cephalosporins and carbapenems in enteric bacteria and non-fermenting pathogens. AmpC type enzymes have been proved to exhibit high levels and high hydrolysis capacity, leading to antibiotic resistance, especially in strains with low permeability [93]. Since 1990, the emergence of AmpC-type enzyme-carrying plasmids has become problematic due to inter-species transfer, increasing the resistance to different BLAs [94].

Although initially considered irrelevant β -lactamases, occasionally encountered [95], carbapenemases became today the principal mechanism of carbapenem resistance in Gram-negative bacteria. Class A carbapenemases, such as the SME enzymes from *Serratia marcescens*, have been identified since 1980 in Europe and America [96]. MBLs were initially identified in Japan, where the first enzyme was IMP [97], and in Italy, where VIM β-lactamase was identified [98]. However, at present, these enzymes are associated with some geographical regions without having a considerable spread [99,100]. Since 2000, KPC-type carbapenemase-encoding plasmids have been identified in many parts of the world, especially in *K. pneumoniae*, the most common being KPC-2 and KPC-3 [101,102], but they may occur in most Gram-negative bacteria. KPC-producing bacteria are associated with high mortality rates, with approximately 51% of infections being caused by colistin-resistant *K. pneumoniae* strains [103]. Another category of carbapenemases associated with several infection outbreaks is class D, also called oxacillinase (CHLD) due to their ability to hydrolyze oxacillin. Over 400 OXA enzymes have been characterized, mostly having the ability to hydrolyze carbapenems. In *A. baumannii*, the presence of OXA-type β-lactamases, which hydrolyze carbapenems, is one of the significant mechanisms of resistance, OXA enzymes such as OXA-23, OXA-24/40, and OXA-58 being among the most prevalent in this species [104,105]. OXA-23 was identified in Scotland [106], later disseminated globally, now reaching a high frequency in *A. baumannii* isolates [107,108]. Genes encoding OXA-type β-lactamases have been identified mainly chromosomally or plasmid located in *A. baumannii* strains [109,110]. In *P. aeruginosa*, carbapenemases were reported in several parts of the world, especially in the case of strains harvested from hospitalized patients. In a cross-sectional study conducted in Iran, 146 strains associated with nosocomial infections were investigated, in which the *bla*_OXA23_ and *bla*_OXA24/40_ genes were identified [111]. Following the investigation of 1969 *P. aeruginosa* strains collected from four hospitals in Dubai, MBL genes such as VIM-2, VIM-30, VIM-31, and VIM-42 were identified [112]. Increasing rates of carbapenem-producing *P. aeruginosa* isolates were reported in an extensive study conducted in Canada, in which 3864 isolates were analyzed. Broad genetic diversity was observed among both carbapenem-resistant and XDR phenotypes of *P. aeruginosa*, with *bla*_GES_, *bla*_KPC_, *bla*_NDM_, *bla*_IMP_, *bla*_VIM_, and *bla*_OXA-48_ encoding genes [113]. Many reports have also highlighted the presence of carbapenemases in *Enterobacter* spp., another category of pathogens belonging to ESKAPE group. Studies have reported the presence of NDM, KPC [114,115], OXA-48 [116,117], VIM, and IMP enzymes [115,118], demonstrating the vast epidemiology of this carbapenem-producing *Enterobacteriaceae*. 

Subsequently, β-lactamases were classified based on functional analysis. One of the best-known classification schemes based on the functional structure is the one proposed by Bush, Jacoby, and Medeiros in 1995. Within this classification, β-lactamases are divided into three groups, depending on the degraded β-lactam substrate and the inhibitors’ effects. The first group includes class C cephalosporinases from the molecular structure classification. The second group comprises β-lactamases other than those from the first group, which have serine at the active site. The third group includes MBLs corresponding to class B of Ambler’s classification [119]. In 2010, Bush and Jacoby expanded the functional classification scheme, with avibactam’s addition differentiating carbapenemases with the active site of serine from MBLs, representing a possible diagnostic marker in phenotypic cellular reactions [120,121]. 

In more recent β-lactamase classification schemes, the classification criterion is the association between three-dimensional structure and functional characteristics, especially in class A/group 2 β-lactamases [122]. Currently, the number of β-lactamases continues to increase almost exponentially due to the possibility of genomes sequencing [123]. However, increasing the number of β-lactamases brings new challenges such as incomplete sequencing of genes declared as encoding for β-lactamases, incorrect annotation, or lack of correlation with function due to lack of expression [124].

## 4. β-Lactamase Inhibitors (BLIs)

Since the introduction of penicillin, the rapid evolution of pathogen resistance to most antimicrobial compounds has remained challenging. The emergence of bacterial resistance to most antibiotics used in therapy has led to the development of new compounds that block β-lactamases involved in resistance. BLIs can be used in combination with antibiotics to prevent their degradation by β-lactamases. Although attempts have been made to improve the action of BLAs, as well as to introduce new generations, the combination of BLAs and BLIs is still an effective strategy to combat β-lactamase-mediated resistance [12]. Since the discovery of clavulanic acid [125] as an inhibitor of most class A β-lactamases, various combinations of penicillins and inhibitors (amoxacillin-clavulanate, ampicillin-sulbactam, piperacillin-tazobactam) have been used to treat infections caused by β-lactamase-producing pathogens [126]. However, these inhibitors’ limited spectrum of action has led to the need to develop compounds with more efficient action and a broader spectrum. One of the most significant categories of recently introduced inhibitors is diazabicyclooctanones (DBOs), with avibactam being the first inhibitor successfully used in the clinic in combination with oxyiminocephalosporin ceftazidime [127]. Avibactam has a bicyclic core structure and can reverse the active site of serine β-lactamases in a reversible manner [128], being a potent inhibitor of class A and C β-lactamases. The combination with ceftazidime has been clinically approved for treating abdominal and UTIs and pneumonia [129]. Success in the use of avibactam in the clinic has led to the introduction of new DBOs alternatives, of which relebactam is in an advanced stage of development in combination with imipenem [130,131,132]. Next, the main β-lactamase inhibitors commonly used in therapy will be described, as well as the new combinations of inhibitors and antibiotics.

### 4.1. Well Documented BLIs

Clavulanic acid and his combinations [co-amoxiclav (combined with amoxicillin) and coticarclav (combined with ticarcillin)] are active against Ambler class A β-lactamases particularly. Clavulanic acid inhibits the plasmid-encoded β-lactamases of *E. coli* and *S. aureus*, but not the chromosomally-encoded variants revealed by *Pseudomonas* and *Enterobacter* strains [133]. Therefore, co-amoxiclav is active against both amoxicillin-sensitive and select amoxicillin-resistant strains belonging to difficult to treat pathogens [134].

Sulbactam and tazobactam are penicillanic acid sulfones with β-lactamase inhibitory activity capable of inhibiting TEM-type β-lactamases, sulbactam being less effective against SHV- and OXA-variants [135]. In *A. baumannii* strains, sulbactam can inhibit PBP3, proving a direct antibacterial activity against this genus [136]. There have been introduced different combinations of sulbactam with BLAs represented by ampicillin-sulbactam (low activity against ESBL-producers belonging to *E. coli*, and *K. pneumoniae* strains [137], cefoperazone-sulbactam (active against *Pseudomonas* spp., *Acinetobacter* spp., *Klebsiella* spp., *E. coli* ESBL-producing strains) [138]. Available combinations of β-lactams and tazobactam are represented by ceftolozane-tazobactam (approved by FDA for the treatment of cUTIs that shows activity against MDR *P. aeruginosa*, ESBL-producing *K. pneumoniae*, and *E. coli* strains) [139]. On the other hand, it has been proved that piperacillin-tazobactam has a higher spectrum of activity against *Pseudomonas* spp., *Klebsiella* spp., *E. coli*, *Enterobacter* spp., and *Citrobacter* spp. ESBL-producing strains compared to cefoperazone-sulbactam and ticarcillin-clavulanic acid [138]. 

Brobactam, structurally very similar to sulbactam and tazobactam, possess a 8–50 fold higher potency than clavulanic acid against chromosomally-encoded cephalosporinase enzymes in *Enterobacteriaceae* and the ampicillin-brobactam combination held a superior in vitro activity to co-amoxiclav against *Proteus vulgaris*, *Morganella morganii*, *Citrobacter freundii*, and *Yersinia enterocolitica* [140]. 

### 4.2. Newer BLIs 

Other BLIs were introduced for the next generation of combined therapy, one such class of newer, non-β-lactam BLIs is represented by the diazabicyclooctanes (DABCOs), based on a (5R)-7-oxo-1,6-diazabicyclo[3.2.1]octan-6-yl sulphate core, of which the approved compounds for clinical use are: avibactam, relebactam, macubactam, zidebactam, and nacubactam (active against MDR Gram-negative rods) and are able to augment the activity of β-lactams in the absence of β-lactamases [141] in a different species including *A. baumannii* [142] and *P. aeruginosa* [131]; WCK 5107, WCK 5153 (a β-lactam enhancer effect against *A. baumannii* [142] and *P. aeruginosa* strains [131]); WCK 4234 and his combination with meropenem called WCK 5999, has been shown to be superior to meropenem monotherapy against MDR clinical isolates of *A. baumannii* [143], including OXA-23- and OXA-24-producing strains, *K. pneumoniae* [144], and *P. aeruginosa* [143]; ETX2514 (a DABCO analogue with class A, C, and broad class D β-lactamase inhibitory activity) [132]; active especially against the class D enzymes OXA-10, OXA-23 and OXA-24 [132], *Enterobacteriaceae* including mcr-1- positive *E. coli*, *E. cloacae*, *Stenotrophomonas maltophilia*, *Citrobacter* spp. and class B β-lactamase-positive and -negative CRE); GT-055 (active against class A, C, D, and some class B β-lactamases, has intrinsic activity against some *Enterobacteriaceae* and is reported to potentiate GT-1 against MDR strains *of A. baumannii* and *P. aeruginosa* strains) [145]; boronic acid transition state inhibitors (BATSIs) a BLI with activity against serine β-lactamases and of the BATSIs—vaborbactam.

In the following paragraphs, the most frequently recommended DABCOs combinations will be presented.

#### 4.2.1. Ceftazidime-Avibactam (CAZ-AVI)

CAZ-AVI is an intravenous combination approved by the FDA and recommended for treating complicated intraabdominal infections (cIAF) in combination with metronidazole, pyelonephritis, and other cUTI, HAP, including ventilator-associated pneumonia (VAP), and other critical diseases triggered by Gram-negative aerobes, in which treatment options are often limited [146]. As avibactam is a non-β-lactam, β-lactamase inhibitor, it brings the advantage of being recycled; thus, after the covalent acylation of β-lactamases, a process that is also reversible, follows the deacylation and the release of avibactam in an integer and fully functional state [147]. Avibactam is potent over class A (KPC-2/3, TEM-1), class C (AmpC-type β-lactamase), and some class D (OXA-10, OXA-48) enzymes, and has no activity on MBLs-producing strains [127,148,149] (Table 1). Its introduction into clinical practice, however, has brought significant advantages over many non-susceptible ceftazidime species, such as some *Enterobacteriaceae* and *P. aeruginosa*; however, its activity on *Acinetobacter* spp., Gram-positive cocci, and anaerobes remains moderate [127].

A study conducted by the International Network for Optimal Resistance Monitoring (INFORM) analyzed more than 34,000 strains of *Enterobacteriaceae* from patients with intra-abdominal, urinary tract, lower respiratory tract, bloodstream, and dermatological infections between 2012–2014. In total, 99.5% of *Enterobacteriaceae* were sensitive to CAZ-AVI following the FDA-indicated microbiological endpoints (susceptible MIC of ≤8 μg/mL; resistant MIC of ≥16 μg/mL). The MICs required for inhibiting 90% of bacterial strains (MIC_90_) for CAZ-AVI was 0.5 μg/mL, lower than the MIC_90_ required for cephalosporin alone (64 μg/mL) to achieve the same yield. Interestingly, of the 185 (0.5%) strains not susceptible to CAZ-AVI, almost a third were MBLs producers (IMP, VIM, NDM) that were also resistant to carbapenems [149]. In parallel, other studies have revealed significant differences in the susceptibility of ESKAPE species to CAZ-AVI. For example, 92% of *P. aeruginosa* strains collected in another INFORM trial were susceptible to this therapeutic combination, requiring an MIC_90_ of 8 μg/mL [150]. In contrast, it was noticed that *A. baumanii* strains of European origin are not susceptible to CAZ-AVI, as MIC_90_ was 64 μg/mL [150]. This therapeutic combination is also not effective against Gram-positive bacteria [146].

The Phase 3 RECAPTURE program, which included two multicenter, randomized, double-blind, double-dummy parallel group-trials, analyzed CAZ-AVI, and doripenem’s comparative efficacy in 1033 pyelonephritis and cUTI patients [151]. Out of the total number of these patients, only 810 were eligible, with 393 and 417 receiving CAZ-AVI and doripenem, respectively. Hospitalized patients were randomized 1:1 to receive CAZ- AVI intravenously 2.5 g every 8 h and doripenem 500 mg every 8 h, requiring slight changes where an impaired renal function was reported. After the first five days of treatment, patients were allowed to receive oral therapy for the next 5 or 9 days until the end of treatment. Interestingly, in more than 95% of the analyzed patients were reported *Enterobacteriaceae* strains and almost 75% were *E.coli*. Of the non-*Enterobacteriaceae* group, *P. aeruginosa* was the most common isolate. The non-inferiority of CAZ-AVI vs. doripenem was validated by FDA co-primary end-points both in terms of a symptomatic resolution reported by the patient on day 5 [276 of 393 (70.2%) vs. 276 of 417 (66.2%) patients (difference, 4.0%)], as well as microbiological eradication in the test of cure [280 of 393 (71.2%) vs. 269 of 417 (64.5%) patients (difference, 6.7%)]. Notably, the safety profile of CAZ-AVI was much better compared to that of cephalosporin given alone; however, no information has been obtained on the effects of these compounds on renal function [151].

Additionally, Shield and collaborators compared the efficiency of CAZ-AVI (*n* = 13) with different regimens based on a carbapenem and an aminoglycoside (CB + AG) (*n* = 25), a carbapenem with colistin (CB + COL) (*n* = 30), and other types of agents in the management of carbapenem-resistant *K. pneumoniae*. Interestingly, CAZ-AVI treatment was much more effective in the clinical setting than the other two agents-based therapeutic regimens (85% vs. 44%, *p* = 0.006). Furthermore, it was shown that CAZ-AVI can improve the overall survival rates at 90 days to 92% versus 56%, 63%, and 49% respectively for patients treated with other therapeutic formulations (CB + AG, CB + COL, others). Overall survival rates were also improved when CAZ-AVI was co-administered with gentamicin vs. its single administration (100% vs. 87.5%). Last but not least, the nephrotoxicity of CAZ-AVI is lower (18%) than that of CB + AG (44%), CB + COL (48%), which makes it suitable for the treatment of carbapenem-resistant *K. pneumoniae* [152].

Other additional studies, such as that of van Duin and colleagues, have confirmed the CAZ-AVI’s therapeutic efficacy in patients with carbapenem-resistant *Enterobacteriaceae*, affected by respiratory or bloodstream infections. Thirty-nine patients received CAZ-AVI, while 99 were treated with colistin. Statistical analyzes revealed that CAZ-AVI was associated with lower causal mortality in hospital at 30 days than colistin (9% vs. 32%, *p* = 0.001) and a 64% higher probability of achieving therapeutic success [153]. Therefore, like other studies, this study potentiates that CAZ-AVI can be a safe and effective therapeutic strategy in treating the most complicated infectious bacteria.

Several studies highlighted some potential mechanisms that can make bacteria refractory to antibiotics regarding the resistance to this combination. As observed in the INFORM trial, the most common mechanism involved in acquiring CAZ-AVI resistance is the production of MBLs that are refractory to avibactam’s action [149]. Mutations in various KPC or AmpC-type enzymes have also been identified as factors that counteract the antibacterial effects of CAZ-AVI [154,155]. Further complicating this scenario, the observation that 41 of the 185 *Enterobacteriaceae* in INFORM are not displaying any metal β-lactamase suggests that other mechanisms are involved in the process of CAZ-AVI resistance. These key determinants may probably include alterations in therapeutic targets (e.g., PBPs), amplified drug efflux, or decreased outer membrane permeability [156].

#### 4.2.2. Ceftolozane/Tazobactam (CEF-TAZ)

CEF-TAZ is a new semisynthetic antipseudomonal cephalosporin used in the treatment of cUTIs, cIAF, and HAP. CEF is an oxyimino-aminothiazolyl cephalosporin very similar structurally to CAZ but has a modified side chain that contributes to his stability in the presence of AmpC β-lactamases, prevents the hydrolysis of the β-lactam ring, and thus confers potent activity against *P. aeruginosa* strains [157]. CEF shows two times higher inhibitory activity and binding affinities for some PBPs (e.g., PBP1b, PBP1c, PBP2, and PBP3) compared to CAZ [158]. On the other hand, TAZ is a β-lactamase inhibitor able to protect the β-lactam against the hydrolysis and inhibits most class A narrow-spectrum β-lactamases, ESBLs, and class C enzymes (Table 1) and enhances the activity of ceftolozane against some ESBL-producing *Enterobacteriaceae* and anaerobes [159]. 

CEF-TAZ has in vitro activity against *Streptococcus* species; however, like ceftazidime, ceftolozane–tazobactam has diminished activity against *S. aureus* strains; improved activity against MDR or XDR *P. aeruginosa* and a significant number of species belonging to *Enterobacteriaceae* family such as *E. coli, K. pneumoniae* (susceptibles at MIC of ≤8 mg/L); *Enterobacter* spp. (MIC50/90, 0.5/8 mg/L), *Citrobacter* spp. (MIC50/90, 0.25/32 mg/L), *Serratia* spp. (MIC50/90, 0.5/2 mg/L), *K. oxytoca* (MIC50/90, 0.25/2 mg/L), and *P. mirabilis* (MIC50/90, 0.5/0.5 mg/L) [159,160]. It has also been demonstrated that CEF-TAZ has in vitro activity against *Bacteroides fragilis, Prevotella*, and *Fusobacterium* spp; however, it has diminished or no activity against other *Bacteroides* spp. and anaerobic Gram-positive cocci [161].

It has been shown that β-lactamases such as TEM-1, TEM-2, SHC-1, and OXA-1 have reduced effect on the activity of CET-TAZ; furthermore, there have been described some ESBLs such as TEM-3–9, SHV-2–4, OXA-2, and CTX-M-3–18 able to reduce the activity of the drug, however remaining efficacious [44,162].

#### 4.2.3. Imipenem/Relebactam (IMI-REL) and Meropenem/Vaborbactam (MER-VAB)

The first β-lactamase inhibitors displaying in vitro activity against class A and C β-lactamases (Table 1) were introduced in combination with carbapenems REL (with IMI) and VAB (with MER) [163]. REL is structurally related to AVI, differing by adding of a piperidine ring to the 2-position carbonyl group [164]. There have been demonstrated that the REL addition reliable reduces the MIC values for IMI and increase IMI susceptibility level in *P. aeruginosa* strains [165,166]. It has been revealed variable susceptibility levels to IMI-REL in carbapenem-resistant *Enterobacteriaceae* (CRE) by different authors: e.g., Canver et al. [167] and Haidar et al. [168], demonstrated 100% susceptibility in *K. pneumoniae* KPC-2 and KPC-3 producing isolates; opposite, Livermore et al. [169] have shown a minimum level of susceptibility in *K. pneumoniae* VIM, IMP, and NDM producing strains.

Several authors evaluated by in vitro studies the IMI-REL activity against *P. aeruginosa* strains and have demonstrated that approx. 94% of the tested isolates revealed susceptibility to IMI- REL [165,166,169,170,171]. Compared to most Gram-negative ESKAPE pathogens, IMI-REL susceptibility levels among *A. baumannii* strains were low [165,166]. For anaerobic Gram-negative species such as *Bacteroides* spp., *Parabacteroides* spp., *Prevotella* spp., *Fusobacterium* spp., *Desulfovibrio* spp, and *Veionella* spp, the IMI-REL susceptibility levels were between 99 and 100% [172].

VAB is a cyclic boronic acid with high affinity to serine β-lactamases, and both can inhibit class A β-lactamases such as TEM, SHV, CTX-M, KPC, class C (AmpC) (Table 1); however, they have not been proven to significantly inhibit class B (e.g., IMP, VIM, NDM) or class D (e.g., OXA24/40) produced by Gram-negative bacilli [163,166,173]. It has been shown that VAB can restore the MER activity, inhibiting the activity of serine β-lactamases [174]. MER–VAB acts against several Gram-negative organisms [144]. It has been established that by VAB addition the activity of MER is restored against CRE isolates producing Ambler class A β-lactamases, such as KPC- and KPC-3 [166]. MER–VAB demonstrated potent in vitro activity in nosocomial *E. coli* isolates co-producing AmpC and KPC [166]. In nonfermenting Gram-negative rods, especially *P. aeruginosa* and *Acinetobacter* spp., the MER-VAB activity is very similar to MEM because of carbapenem resistance in *P. aeruginosa* and *Acinetobacter* spp. can be the result of several mechanisms that would not be impacted by VAB addition, including reduced outer membrane permeability (commonly due to the loss of the OprD porin channel), overexpression of efflux pumps (particularly MexAB-OprM or MexEF-OprN), and production of MBLs [175] or class D β-lactamases in *Acinetobacter* spp. [175,176]. There is scarce information regarding the activity of MER-VAB against Gram-positive bacteria and anaerobic bacteria, but it would be expected that the anaerobic activity of MER-VAB should be similar to that of MER alone, considering that MER is active against methicillin-sensitive *S. aureus* (MSSA), *Streptococcus pyogenes*, *S. agalactiae*, penicillin-sensitive *S. pneumoniae*, and some strains of *E. faecalis* and *E. faecium* [174] and also against several anaerobic bacteria, including *B. fragilis* and *Fusobacterium* spp. [177].

#### 4.2.4. Cefepime/Zidebactam (WCK 5222)

WCK 5222 contains a BLI (zidebactam) and a fourth-generation cephalosporin (cefepime). WCK 5222 revealed in vitro antimicrobial activity against *Enterobacteriaceae*, *P. aeruginosa* [178], and *A. baumannii* strains [179]. Currently, this combination is in a clinical development program to treat MDR infections caused by Gram-negative bacteria. Zidebactam is a non-β-lactam bicyclo-acyl hydrazide that acts either by direct inhibition of β-lactamases or by inhibition of PBP2 [142]. It is considered a broad-spectrum inhibitor of action against all four β-lactamase classes (A, B, C, and D), although the action on MBLs is not recognized. Zidebactam binds with a high affinity to PBP2, while cefepime has a high affinity for PBP3 and a lower affinity for PBP2 and PBP1a/1b. This inhibitor acts by improving the antibiotic’s action by complementary binding to PBPs [131]. Regarding clinical trials, Phase I clinical trials have already been performed that have analyzed the efficacy, safety, and tolerability of these compounds by intravenous administration to healthy adult patients (ClinicalTrials.gov registration no. NCT02674347 and NCT02707107). Rodvold et al. conducted a clinical study in 36 patients in which they analyzed WCK 5222 levels in plasma, epithelial-lining fluid, and alveolar macrophage. Following intravenous administration of WCK 5222, moderate adverse reactions were observed in three patients. In general, the administration of WCK 5222 in seven doses proved safe and well tolerated by subjects. The concentration of zidebactam and cefepime in alveolar macrophage persisted 10 h after administration, demonstrating the possibility of using this combination to treat nosocomial pneumonia [180]. The effects of WCK 5222 were analyzed in a neutropenic mouse *A. baumannii* lung infection model. The cefepime MIC against these strains ranged from 2 to 16 mg/L, suggesting a lack of significant expression of FEP-impacting β-lactamases. The addition of ZID did not lower the MIC of FEP against any of these *A. baumannii* strains. However, time-kill studies revealed that ZID mediated the enhancement of bactericidal activity at sub-MICs of FEP. This study revealed that ZID exerted a reduction in the MIC of FEP, and in combination with the high FEP-ZID clinical doses selected, this feature could help provide consistent clinical effectiveness even for the problematic challenging patients, such as those with reduced drug exposures [181]. The activity of WCK 5222 was investigated both in vitro and in vivo, in a neutropenic and pneumonia mouse infection model, against *K. pneumoniae* [182], *A. baumannii* [183], *P. aeruginosa* [184,185], and *Enterobacter* spp. [186].

#### 4.2.5. MBL Inhibitors (MBLi)

The clinical introduction of DBOs and vaborbactam has broadened the spectrum of options for treating nosocomial infections caused by MDR Gram-negative bacteria. However, concerning MBLs, none of these inhibitors exerts effective action, thus increasing the need to develop inhibitors that specifically target MBLs. Currently, no inhibitors of MBLs have been approved for use in the clinic. The development of MBLs inhibitors has focused on compounds that bind and/or chelate zinc ions within the active enzyme site [187,188]. Aspergillomarasmine A, a fungal compound active against the MBLs NDM-1 and VIM-2, acts by chelating and removing the active site zinc ions and can re-sensitize to MER the *Pseudomonas* spp., *Acinetobacter* spp., and *Enterobacteriaceae* MBL-producing strains [134]. Another category of inhibitors that act by binding to zinc ions within the enzyme site is thiol-based compounds such as bisthiazolidines and small bicyclic compounds with inhibitory activity against B1, B2, and B3 MBLs [189]. Phosphonate-containing compounds (6-phosphonomethylpyridine-2-carboxylates) are another category of compounds whose action against B2 and B3 MBLs has been reported. In vitro analyses have shown that these compounds interact with zinc ions in the enzyme active site [190].

Boronate compounds represent a new category of compounds with promising activity, especially on MBLs. The boron feature to adopt a tetrahedral geometry gives it the ability to mimic the tetrahedral species formed during hydrolytic reactions [191]. This property allows the use of these compounds both as inhibitors and in the study of the mechanism of action of β-lactamases. This is due to mimicking the tetrahedral transition of oxyanions in acylation or deacylation reactions during β-lactams’ hydrolysis [192]. Currently, taniborbactam (VNRX-5133), a bicyclic boronate, is in phase 3 clinical testing in combination with cefepime to treat UTIs [193,194,195,196]. A new concept in the development of MBLs inhibitors involves obtaining compounds that bind to highly conserved active sites of the Lys224 type within B1 MBLs [197] or the Cys221 site within NDM-1 (ebselen compound) [198]. Recently, these two concepts have been combined to form a dual inhibitor that binds to both Lys224 and Cys221 to obtain a broader spectrum of action against B1 and B2 MBLs subclasses [199].

Although there are studies that have demonstrated the action of some compounds against MBLs, finding effective inhibitors with a spectrum of action encompassing the MBL superfamily remains a challenge that must be considered in future studies.

## 5. Alternative Approaches to Combat ESKAPE Pathogens

### 5.1. Antimicrobial Peptides (AMPs)

With a large activity spectrum including protozoa, bacteria, archaea, fungi, plants, and animals, AMPs (amphipathic molecules containing about 11–50 amino acid residues) may represent an alternative to current antibiotics against ESKAPE pathogens [200], acting by interaction with cell membrane through electrostatic interactions and causing the inhibition of protein and nucleic acid synthesis, and final cellular lysis [201,202]. The diversity of AMPs (natural or bioengineered) makes them attractive candidates against ESKAPE pathogens in clinical studies. However, further studies and tehcnologies are required to improve the in vivo efficiency and stability of AMPs, and therefore, to increase the specificity against the infectious agent and decrease cytotoxicity to mammalian cells. The diversity of AMPs (natural or bioengineered) makes them attractive candidates against ESKAPE pathogens in clinical studies (Table 2).

#### Resistance to AMPs

Similar to the conventional antibiotics another challenge is represented by the fact that bacteria developed resistance against AMPs by alteration of the bacterial cell surface or by the release of proteolytic enzymes, which results in the hydrolysis of the AMPs, for e.g., the proteases released by *Enterobacteriaceae* included in the PhoPQ, PmrAB, and RcsBCD Phosphorelay system or elastases in *P. aeruginosa* [236]; the *K. pneumoniae* capsule stops the AMPs entrance. There have been several nanocarriers developed—such as novel polymeric and lipidic nanoparticles, carbon nanotubes, micelles, liposomes, ethosomes, aquasomes, transferosomes, niosomes, catezomes, pharmacosomes, cubosomes, polymersomes, microspheres, dendrimers, nanocapsules, for delivering the AMPs, which may help in avoiding the low bioavailability, proteolysis, or susceptibility and toxicity associated with APMs [237,238].

### 5.2. Metal Nanoparticles

Metal nanoparticles (MNPs) represent an alternative to current antibiotics due to their activity against ESKAPE pathogens [239] and include NPs containing Ag, Au, Zn, Cu, Ti, Mg, Ni, Ce, Se, Al, Cd, Y, Pd, or superparamagnetic Fe [240]. MNPs can interfere in the metabolic activity of a bacterial cell [241], penetrate the biofilms and inhibit the biofilm formation [242]. NPs can act at the level of cellular wall causing changes in cell membrane permeability or across the bacterial membrane and interact with intracellular targets, leading to macromolecular structures and functions alteration, oxidative stress, or electrolyte balance disorders [243]. The advantages of the most known MNPs against ESKAPE pathogens, and their mechanisms are shown in Table 3.

### 5.3. Bacteriophages

Shortly after their reporting by Twort in 1915 [268] and d’Herelle in 1917 [269], bacteriophages began to be used to treat bacterial infections. Bacteriophages are able to infect bacteria by detecting surface receptors, injecting their genetic material into the host, and replicating using the host cellular machinery [200]. The isolation of lithic phages from the hospital sewage indicated their use as therapeutic agents against MDR ESKAPE pathogens [270]. Bacteriophages used in the treatment of bacterial infections have several advantages such as high specificity, preventing damage to normal microbiota and eukaryotic cells, rapid proliferation in the bacterial host, low doses required for treatment [271]. Also, unlike antibiotics, phages do not loose their activity following mutations acquired inside the host [272].

The bacteriophages’ efficiency against ESKAPE pathogens has been demonstrated by in vitro and in vivo studies in animal models or in treated patients, having been shown to reduce the mortality rates and speeding the healing process. Promising results have been obtained for eye infections with VRSA (vancomycin-resistant *S. aureus*) [273], pancreatitis [274], diabetic ulcers [275], or UTIs [276,277]. Several other clinical studies have been performed recently, the top results being summarized in the Table 4. Starting from the promising studies performed both in vitro and in vivo, in animal models, a series of commercial kits to prepare beech suspensions with action against ESKAPE species have been developed. Examples of such commercial kits are “Pyophage”, “PhagoBioDerm”, “Sextaphage”, and “Staphal”. Pyophage (Georgian Eliava Institute of Bacteriophage, Microbiology, and Virology) contains bacteriophages that act against bacteria involved in pyoinflammatory and enteric diseases. PhagoBioDerm is a bandage-type polymeric structure impregnated with a cocktail of phages, antibiotics, and other active substances to treat ulcers and infections caused by *S. aureus* and *P. aeruginosa* [278]. Sextaphage (Microgen, ImBio Nizhny Novgorod, Russia) is a cocktail used against *P. aeruginosa* and *E. coli*, and Staphal (Bohemia Pharmaceuticals, Slovakia) is an antistaphylococcal beech. These kits’ clinical potential was subsequently studied either in model animals or in the clinic to determine the spectrum of activity against bacterial strains. However, phage therapy has several limitations. Its high specificity is one of them. In order to surpass it, cocktails containing more phages, each acting on a particular bacterial species is designed [279] to extend the spectrum of action [280]. Determining the safety of phage therapy is another issue requiring careful genomic characterization. Phages used in therapy should not contain resistance or virulence genes or elements involved in the transfer or integration of these genes into the host bacterial genome, such as site-specific integrases or recombinases, in order to prevent the HGT of virulence genes or antibiotic resistance genes [281]. Also, phages should not elicit an immune or allergic response [282,283]. Another limitation refers to the phages’ stability and their proper administration to have the expected effect at the site of infection. In therapy, phages can be administered orally, nasally, topically, or powdered formulations [284,285]. Studies have also shown improved efficacy of phages when administered in combination with liposomes [286].

### 5.4. CRISPR Cas—An Emergent Strategy in Controlling ESKAPE Pathogens

The use of CRISPR/Cas strategy for combating bacterial resistance is one of the most exciting approaches to fight ESKAPE pathogens.

The CRISPR/Cas acts as a bacterial ‘immune’ system that can detect and degrade foreign nucleic acids through the activation of caspases. CRISPR/ Cas system has a high specificity, provided by short repetitive sequences, located in CRISPR loci, and separated by sequences of 26–72 base pairs derived from MGEs [308]. The action of the CRISPR system against foreign genetic material occurs in three stages: (i) acquisition, in which single sequences (spacer) derived from MGEs and delimited from each other by repetitive sequences are taken up in the repetitive loci from the host chromosome; (ii) expression, in which the repetitive and spacer sequences are transcribed into a single RNA transcript that will be afterward cleaved by caspases into small CRISPR RNA; and (iii) interference, in which the complementarity between CRISPR RNAs and foreign nucleic acids allows the recognition and degradation of foreign DNA by caspases [309,310]. The distinction between self and non-self is possible due to protospacer sequences derived from foreign nucleic acids, which are flanked by protospacer adjacent motifs (PAMs). The target recognition is achieved only by identifying these sequence motifs not stored in CRISPR loci, thus eliminating the danger of degradation of the own nucleic acid [310]. 

It has been shown that CRISPR system is limiting the plasmid entrance into bacterial cells, a feature that could be further exploited for the limitation of antimicrobial resistance transmission by HGT [311]. CRISPR system has been used for *A. baumannii* genome editing by introducing insertions, deletions, and point mutations in the oxidative stress (OxyR) gene, for increasing the sensitivity of *A. baumannii* strains to oxidative stress [312]. Also, CRISPR technology was used to to increase the susceptibility of different *Enterobacteriaceae* by successfully decreasing the number of plasmid carrying the *bla*_TEM-1_ gene [313].

In *K. pneumoniae*, Sun et al. designed the pCasKP-pSGKP editing system to obtain the deletion of the tetA and ramR genes associated with tigecycline resistance and of the mgrB gene associated with colistin resistance [314]. Similarly, Wang et al. built a two-plasmid system, pCasKP-pSGKP, to achieve the deletion of the dhaF, pyrF, fepB, ramA, fosA, pyrF, fepB, and ramA genes in two clinical *K. pneumoniae* isolates [315].

More recently, Hao et al. built a CRISPR system (pCasCure) that was electrotransferred to various CRE isolates—including *K. pneumoniae*, *E. coli*, and *E. hormaechei*—in order to perform the deletion of KPC, NDM, and OXA-48 carbapenemases. The authors obtained the deletion of the specific genes with an efficiency percentage of over 94%. It has also been observed that the pCasCure system can be used to eliminate endemic plasmid types that confer resistance to carbapenems, such as *bla*_KPC_-harboring IncFIIK-pKpQIL and 35 IncN pKp58_N, *bla*_OXA-48_-harboring pOXA-48-like and *bla*_NDM_-harboring IncX3 plasmids [316].

In *S. aureus*, one of the primary pathogens from the ESKAPE group, numerous studies have demonstrated the effectiveness of the CRISPR system in deleting ARGs and eliminating plasmids carrying ARGs. Bikard et al. designed a CRISPR technology by inserting the CRISPR array in a staphylococcal vector to obtain pDB114, programmed to target kanamycin and methicillin-resistant genes. The authors obtained sequence-specific killing of kanamycin and methicillin-resistant staphylococci, loss of pUSA02 plasmid, and staphylococci immunization against pUSA02 transfer [308]. Liu et al. constructed a pLQ-Pxyl/tet-cas9-Pspac-sgRNA system to target the tgt gene and f pLQ-KO-tgt-50 bp plasmid. These experiments revealed the efficiency of CRISPR technology in acquiring successful gene editing in *S. aureus* [317,318].

**Figure 4:**
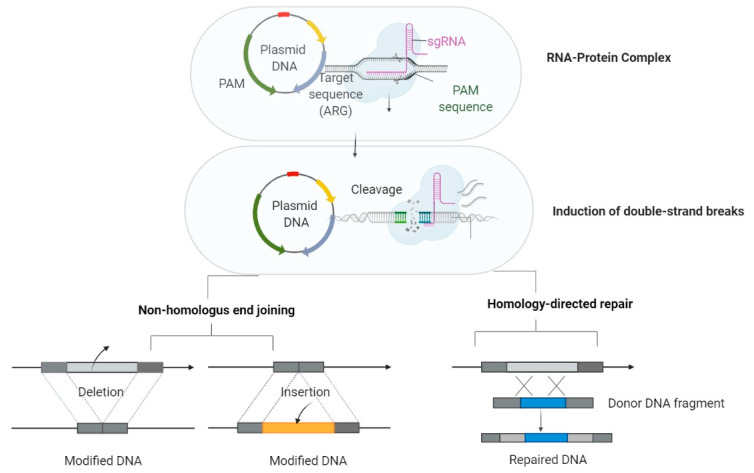
CRISPR Cas9 system targeting MGEs as a powerful tool for genomic editing. The Cas9-sgRNA complex recognizes complementary genetic sites with the 5′ end of the sgRNA. The target gene contains a protospacer, immediately followed by an Protospacer Adjacent Motif (PAM), which is mandatory for the recruitment of the CRISPR Cas9 complex. Cas9 is a dual RNA-guided DNA endonuclease that cleaves each of the two strands three nucleotides upstream of the PAM. Subsequently, several DNA repair mechanisms are employed, such as Non-Homologous End Joining (NHEJ) or Homology Directed Repair (HDR), leading to mutations or gene changes, respectively. CRISPR cas9 system can remove some of the key determinants of antibiotic resistance in bacteria, which is why its use has grown spectacularly in recent years. Figure created with https://biorender.com/.

As with the other species from the ESKAPE group, several studies have sought to program the CRISPR system to study antibiotic resistance mechanisms and remove resistance genes or plasmids by genomic editing in *P. aeruginosa* (Figure 4)*,* a major human pathogen responsible for severe infections in immunocompromised patients or with various conditions such as cystic fibrosis, burns, and cancer [319]. Deletion or mutation experiments on the resistance genes mexB, mexF, mexT, and gyrA, encoding for efflux pumps or for DNA gyrases in *P. aeruginosa* has been achieved with the CRISPR system [318,320]. 

### 5.5. Vaccination

One of the most important pillars in the fight against antimicrobial resistance is vaccination, contributing to reducing antibiotics consumption, the insurgence of resistant serotypes, infection rate with resistant strains and to herd immunity [321]. Multiple trials are currently being conducted both in vitro and in vivo in animal models or in clinical trials to discover feasible vaccines against pathogens, especially those from the ESKAPE group. 

Among the research directions for vaccines are inactivated whole cells (IWC) [322], outer membrane vesicles (OMVs) [323], outer membrane complex (WTO) [324], and several outer membrane proteins including OmpA [325]. Some of the most used components in studies on vaccines’ development against resistant pathogens are OMVs. These components are highly immunogenic spherical structures that contain membrane proteins obtained from the supernatant following centrifugation and/or ultracentrifugation of the cell culture [326] or using detergents to increase the production of OMVs [327,328].

Several studies have recently analyzed in vivo the potential of these components as a vaccine against infection with *A. baumannii* and *P. aeruginosa* strains. Following the intramuscular and intranasal administration of OMV-based vaccine, a decrease of bacterial load and the induction of specific IgG and sIgA responses were observed [329]. After subcutaneous administration of the OprF antigen in Swiss albino mice, active immunization with the production of specific IgG1 and IgG2 antibodies was obtained. Immunization with recombinant protein from *P. aeruginosa* has also been observed to show cross-reactivity against OprF-producing *A. baumannii* isolates. Using serum from mice immunized with this protein, intense bactericidal activity was observed against *A. baumannii* strains [330]. Vaccines built on recombinant proteins have also been developed against *S. aureus* using extracellular bacterial vesicles coating mesoporous silica nanoparticles [329,331,332]. In *K. pneumoniae*, the in vivo studies in mice model infection and non-human primate model of severe lower respiratory tract infection revealed the unique immunogenic properties of T cell-specific epitopes [333], recombinant protein vaccine [334], and polysaccharide capsule type 2 vaccine [335].

There are a limited number of clinical trials aimed at evaluating vaccines against ESKAPE pathogens. The phase I/II randomized trial study used a capsular polysaccharide vaccine serotypes 5 and 8 conjugated to the nontoxic mutant form of diphtheria toxin (CRM197), a recombinant mutant clumping factor A (ClfA), and a recombinant manganese transporter C (MntC), named SA4Ag to achieve immunity against *S. aureus*. This vaccine’s administration in adults aged 65–80 years was well tolerated, inducing antibody synthesis and supporting immune responses 12 months after vaccination [336]. In a recent study by the same research group, this vaccine was administered in a trial with 440 participants. The persistence of immune responses was observed at 36 months after vaccination [337]. The in vivo effectiveness of vaccines have recently been revealed in carbapenem-resistant *K. pneumoniae* [338], using a semi-synthetic glycoconjugate, *P. aeruginosa*, using outer membrane proteins [339], and *A. baumannii*, using a live attenuated *A. baumannii* strain deficient in thioredoxin [340]. 

The majority of currently available bacterial vaccines protect by inducing pathogen-specific antibodies. Therefore, harnessing the antibody component of a potent human humoral response to disseminated infection is valuable for identifying novel protective antigens. This new approach, termed reverse vaccinology 2.0 (RV 2.0), relies on the isolation and recombinant expression of the variable regions of heavy (VH) and light (VL = κ or λ) chain genes of immunoglobulin (focus has centered on IgG) using a variety of molecular tools [341]. Enriched by the development of high-throughput technologies, the screening of large numbers of antibody-secreting cells (ASCs) is also advancing knowledge of host–pathogen interactive biology and auto-immunity. Although this approach has been exploited for viral pathogens, it is expected that the same technologies may also be applied to bacterial pathogens. Growing knowledge in this field could lead to the rational design of new antigens more stable and elicit a high level of functional antibodies.

## 6. Conclusions

BLAs remain at present one of the most potent antibiotic classes against MDR pathogens. Third generation penicillins (aminopenicillins, carboxypenicillins), the fifth generation of cephalosporins, and newly added cefiderococol are the most effective BLAs e against MDR Gram-negative species. Together with the discovery of novel antibiotics from this class, counteracting antimicrobial resistance through BLIs is a promising strategy that could amplify these antibiotics’ action against ESKAPE pathogens. Clinical trials have revealed that some of the most potent formulations in the fight against MDR carbapenemase producing *Enterobacteriaceae* are CAZ-AVI, IMI-REL, and MEM-VAB. However, further studies in establishing new potent inhibitor formulations and their validation in clinical trials are required. Some alternatives against ESKAPE pathogens may be represented by AMPs, phage therapy, nanoparticles, CRISPR/Cas technology, and vaccination. However, their application to date is predominantly at research level and at best at the preclinical setting, with limited number of clinical trials aiming to evaluate these strategies. In this protracted fight against ESKAPE pathogens, the scientific community should assume the role of the defender and design hybrid strategies by combining materials design, nanotechnology, immunity research, and other disciplines, aiming at keeping problematic bacteria under its control. 

## Figures and Tables

**Table 1:** β-lactamase classes susceptibility to the inhibitor’s action.

Agent(s).	Class A	Class B	Class C	Class D
CAZ-AVI				
MER-VAB				
IMI-REL				
CEF-TAZ				
Cefiderocol				

Red—susceptibility; yellow—moderate susceptibility; white—no susceptibility.

**Table 2:** AMPs active against ESKAPE pathogens.

AMPs	Main Activity	Other Effects	Animal Models	References
HLR1–human derived lactoferin peptide	in vitro—microbicidal effect against *S. aureus*	anti-inflammatory properties non-cytotoxic effect	mice, rats, and pig skin infected with *S. aureus*	[203]
Lactoferrin and Lactoferrin derived AMPs	in vitro—antibacterial activity against *E. coli*, *S. aureus*, *Acinetobacter* spp., *P. aeruginosa*	anti-biofilm against *P. aeruginosa* strains	mice	[204]
Brevinin-2Ta (B-2Ta)	in vitro—antimicrobial activities against *S. aureus*, *E. coli*	low cytotoxicity inflammatory effect in vivo using *K. pneumoniae*-infected Sprague-Dawley rats	rats	[205]
DPK-060 structurally derived from human protein kininogen	in vitro—antimicrobial activity against *S. aureus* including MRSA		ex vivo pig skin in vivo—mouses	[206]
Histatin 5—human salivary AMP	in vitro—antibacterial activity against *S. aureus, A. baumannii, E. cloacae, K. pneumoniae* and *P. aeruginosa*	anti-biofilm activity		[207]
Feleucin-K3 AMP and his analogue FK-1D	in vitro antimicrobial activity against *P. aeruginosa*	low-toxicity anti-biofilm activity	in vivo against clinical infections caused by *P. aeruginosa*	[208]
K11 hybrid AMP			in vivo—antimicrobial activity against *A. baumannii*-infected wounds (murine excision)	[209]
(P)ApoBL and r(P)ApoBS—Apolipoproin B human defence AMPs	in vitro antimicrobial activity aginst MRSA and *P. aeruginosa*	anti-biofilm activity anti-inflamatory activity	murine	[210]
Bip-P113 [Bip: β-(4.4′-biphenyl)alanine] AMP	in vitro antimicrobial activity against *S. aureus* and *E. faecium*			[211]
LL-37, a 37-residue AMP derived from human cathelicidin and his derivate FK-16 titanium coated	in vitro antimicrobial activity against ESKAPE patrogens particularly microbicidal effect on *P. aeruginosa*, MRSA and *A. baumannii*	anti-adhesion anti-biofilm activities against *S. aureus*, *P. aeruginosa*, and *A. baumannii*	mice model	[212,213]
Cathelicidin-BF	in vitro antimicrobial activity against *S. aureus* and *P. aeruginosa*	low hemolytic activity on red blood cells; therapeutic potential against acne vulgaris		[214,215]
hBD-3-human-β defensin 3; AMP-29- a sheep myeloid peptide; rCRAMP- a rat cathelin-derived AMP; BMAP-27- a bovine myeloid AMP- 27	in vitro microbicidal activity against *A. baumannii, P. aeruginosa*, and *MRSA*	anti-biofilm activity anti- immunomodulatory activity		[216,217,218]
Indolicidin	in vitro bactericidal activity against *P. aeruginosa* and *S. aureus*			[219]
PMX-30063 (brilacidin)	in vitro bactericidal activity against *S. aureus*			[220]
POL7080 (murepavadin)	in vitro antimicrobial activity against MDR and XDR *P. aeruginosa*			[221]
LTX-109 (lytixar)	in vitro bactericidal activity against *S. aureus*		mouse skin infection model	[164]
chionodracine-derivatives AMPs	in vitro bactericidal activity against *K. pneumoniae, A. baumannii,* MRSA and *P. aeruginosa*			[222]
Ribonuclease 7 AMP	in vitro antimicrobial activity against *P. aeruginosa, S. aureus,* and VRE			[223]
Chrysophsin-1 isolated from the gill cells of *Chrysophrys major*	in vitro antimicrobial activity against MRSA	antiendotoxin properties		[224]
Arenicins-1 isolated from *Arenicola marina* and one of his variants Ar-1[V8R]	in vitro antimicrobial activity against *P. aeruginosa, K. pneumoniae* and *S. aureus*	Ar-1[V8R]—cytotoxicity against mammalian cells		[225]
Pardaxins isolated from mucous glands of *Pardachirus marmoratus*	in vitro antimicrobial activity against *S. aureus, A. calcoaceticus* and *P. aeruginosa*			[226]
Phosvitin from zebrafish	in vitro antimicrobial activity against *S. aureus*	immunomodulatory activity; non-cytotoxic and non-hemolytic	mice model	[227]
Mytimacin-AF, isolated from marine mollusks	in vitro antimicrobial activity against *S. aureus* and *K. pneumoniae*			[228]
PT-3 *Populus trichocarpa* crude extract derived AMP	in vitro antimicrobial activity against *S. aureus*		in vivo antibacterial activity in *S. aureus* infected *G. mellonella* model	[229]
Thanatin and its analog, S-thanatin	in vitro antimicrobial activity against *K. pneumoniae*	low hemolytic activity	mice model	[230]
Pexiganan—a synthetic analog of magainin isolated from *Xenopus laevis*	in vitro bactericidal effect against *P. aeruginosa*			[231]
SET-M33 a synthetic AMPs (similar with colistin regarding the mechanism of action)	in vitro microbicidal activity against *P. aeruginosa* and *K. pneumoniae*	anti-inflammatory and immunomodulatory activities	mice model	[232]
Oritavancin, a synthetic selectively targeted AMPs	bactericidal effects against MRSA and VRSA	anti-biofilm activity		[233]
WLBU2—engineered cationic AMP and his D-enantiomers (D8)	in vitro antimicrobial activity against *A. baumannii* and *P. aeruginosa*	anti-inflamatory activities	mice model	[234]
Oct-TriA2 (2,8-D-Orn, 7-Orn) and Oct-TriA1 based on the tridecaptins	antimicrobial activity against *A. baumannii*, *K. pneumoniae*, and *E. cloacae*	Oct-TriA1 lower haemolytic activity		[235]

**Table 3:** MNPs against ESKAPE pathogens—antimicrobial activity, mechanism of action, and advantages

MNPs Type and Mechanism of Action (MOA)	Agent Used	Targeted Microorganisms and Advantages	References
**Silver (Ag) NPs: MOA**—inhibition of peptidoglycan synthesis, structural modification in the membrane permeability, reactive oxygen species (ROS) generation, lipid peroxidation, interaction with DNA affecting DNA’s replication and finally the cell death	AgNPs-microfibrillated cellulose biocomposite	in vitro antimicrobial activity against *S. aureus* and *P. aeruginosa*	[244]
	Phenolics-coated AgNPs	in vitro antimicrobial effects against *P. aeruginosa* and *Enterobacter aerogenes*	[245]
	Ag nanoform complexed with amorphous TiO_2_	in vitro antimicrobial activity against *S. aureus* and *K. pneumoniae*	[246]
	Ag-containing Hydrofiber^®^ dressing and nanocrystalline Ag-containing dressing	in vitro antimicrobial activity against MRSA and VRE	[247]
	AgNPs immobilized on the surface of nanoscale silicate platelets (AgNP/NSPs)	in vitro antimicrobial activity against MRSA	[248]
	AgNPs from Phyllanthus amarus extract	in vitro antimicrobial activity against MDR *P. aeruginosa*	[249]
	Fungal biosynthesis of AgNPs	antibacterial activity against *S. aureus*; nontoxic, safe, inorganic agent.	[250]
	TiO_2_ nanotubes covered with AgNPs	enhanced antimicrobial activity of the bone/dental implants against *S. aureus;* >80% biocidal activity	[251]
	*Calligonum comosum* and *Azadirachta indica* leaf extracts as stabilizing AgNPs	antibacterial ability against *P. aeruginosa* and *S. aureus*, by causing apoptosis	[252]
	AgNPs synthetized using Ajuga bracteosa extract	bactericidal activity against *K. pneumoniae*, *S. aureus*, and *P. aeruginosa*; antioxidant potential effects; pharmacological importance	[253]
**Cu/Ag NPs**	Graphene oxide/Cu/Ag NPs	in vitro bactericidal activity against *P. aeruginosa, K. pneumoniae*, and MRSA	[254]
**(Golden) AuNPs less toxic than Ag**	AuNPs functionalized with ampicillin	in vitro bactericidal activity against *P. aeruginosa* and *E. aerogenes*	[255]
	Pyrimidinethiol-modified AuNPs	in vitro antimicrobial activity against MDR *E. faecium*, *P. aeruginosa,* MRSA, *K. pneumoniae, A. baumannii*	[256]
	CGNPs (cinnamaldehyde immobilized on AuNPs)	in vitro and in vivo antibiofilm of MRSA and *P. aeruginosa*	[257]
	6-aminopenicillanic acid-coated AuNPs doped into electrospun fibers of poly(ε-caprolactone)	in vitro and in vivo antimicrobial activity against MDR *K. pneumoniae* infections	[258]
	Metallopolymer-antibiotic bioconjugates on AuNPS	antimicrobial activity against *K. pneumoniae* and *S. aureus*	[259]
	AuNPs	in vitro and in vivo bactericidal activity against mastitis-causing *S. aureus*	[260]
**Metal oxide NPs**			
**ZnO NPs**—ROS generation; bactericidal effect, by disrupting the cell membrane; glycolysis and transmembrane proton translocation inhibition	ZnO	antimicrobial activity against MRSA and *P. aeruginosa*; anti-biofilm formation and production of quorum-sensing- in *P. aeruginosa*; anti-biofilm formation MRSA	[261,262]
**Nitric oxide (NO)**— RNS generation	NO-releasing NP	in vitro antimicrobial activity against MRSA, *A. baumannii*, *K. pneumoniae*, and *P. aeruginosa*	[263]
	NO-releasing silica NPs	in vivo bactericidal activity against intracellular *P. aeruginosa* in L929 mouse fibroblasts	[264]
**Cobalt oxide NPs**—oxidative mechanisms< membrane permeability changes; inhibition of DNA replication	Co_3_O_4_	in vitro antimicrobial activity against S. *aureus*	[265]
	Bis hexa decyl trimethyl ammonium cobalt tetrachloride	antimicrobial activity against MDR *S. aureus*	[266]
**Fe_2_O_3_ NPs**—affect the functionality of porin pumps; occupy the active sites of MBLs	Functionalized Fe_2_O_3_ NPs with antibiotics	inhibition growth of *P. aeruginosa*; reducing overcoming resistance and acute toxicity; low cost; synergistic effects with antibiotics	[267]

**Table 4:** Bacteriophages against ESKAPE pathogens

Phage	Targeted Bacteria	Type of Study	Model Application	In vivo Efficacy; Advantages and Survival of Host	Route of Administration	References
Phage ENB6 and C3 (A2 morphotype group)	Ef	in vivo	Murine bacteremia model	Immunocompatible; 100% survival with multiple doses	Intraperitoneal (IP)	[287]
Cocktail of *E. coli* phage ECP311, *K. pneumoniae* phage KPP235, and *Enterobacter* phage ELP140	K & E	in vivo	*Galeria mellonella* infection model	100% reduction after 5 doses; 90% survival	-	[288]
*Enterococcus* phiEF24C, phiEF17H, and phiM1EF22 phages	E	in vitro	-	Inhibition of growth	Co-culture with phages mixture	[289]
phage ϕEf11/ϕFL1C(Δ36)^PnisA^	E	in vitro	-	10–100-fold decrease in viable cells (CFU/biofilm); biofilm eradication	Inoculation with phage	[290]
anti *E. faecium* EFDG1 phage	Ef	ex vivo	Human root canal model	5-log growth reduction in stationary cultures; reducing 2-week old biofilm	-	[291]
vB_SauM_LM12, vB_EfaS_LM99 and vB_EcoM_JB75	S	ex vivo	orthopaedic implant infection model	Great antimicrobial activity; growth reduction	Paper strip	[292]
2003, 2002, 3A and K phage cocktail	S	in vivo	Ventilator-associated pneumonia rat model	Reduced lung damage; 100% survival at 12 h after infection; 58% survival until the end of the experiment	Intravenous (IV)	[293]
Phage coated implant	S	in vivo	Murine model of joint infection	Normal locomotor activity by 10 day; decreasing bacterial adherence	K-wire implant delivery system	[294]
SATA-8505 (ATCC PTA-9476)	S	in vivo	65-year-old woman with Corneal abscess	stabilization of ocular signs; pathogen eradication	Topical (eye drops and nasal spray) and intravenous (IV)	[273]
Staphylococcal phage Sb-1	S	in vivo	Case series (human subjects with diabetic foot ulcer)	Wound healing within 7 weeks	Topical	[275]
*Myoviridae* bacteriophages (AB-SA01)	S	in vivo	Human single-arm non-comparative trial (13 patients)	8/13 patients showed clinical improvement; 5 patients died within the first 28 days	IV	[295]
vB_KpnP_KL106-ULIP47; vB_KpnP_KL106-ULIP54; vB_KpnP_K1-ULIP33;	K	in vivo	*Galleria mellonella* larvae infection model	Mortality rate reduced with 20% upon treatment with phage	Phage inoculation	[296]
*K. pneumoniae* isolated phage	K	in vivo	Case series (48 patients with nonhealing chronic wounds)	significant decrease in the mean depth of the wound; improved score of epithelialization; 39/48 patients had a complete cure	Topical	[297]
*Klebsiella* myPSH1235 and *Enterobacter* myPSH1140 phage	K & E	in vitro	-	Strong bactericidal activity; bacterial density reached to 0 with no viable cells at 24 h after infection	Incubation with phage	[298]
*K. pneumoniae* bacteriophage	K	in vivo	Swiss albino mouse model	gradual reduction of colony-forming unit; complet eradication after 6 days of treatment	Oral	[299]
KpJH46ø2	K	in vivo	Case study (62 year-old diabetic man with prosthetic knee infections)	The restraining of local symptoms, signs of infection, and recovery of function	IV	[300]
Lytic bacteriophage	K	in vivo	Case study (57-year patient with Crohn’ disease)	Bacterial eradication	Oral Intrarectal	[301]
Phage PEV20	P	in vivo	Murine infection model	5-log reduction of bacterial cells	Intranasal; Intratracheal	[285]
US Navy library of bacteriophages	P	in vivo	Case study (2-year-old patient with Di George syndrome)	Bacterial eradication after phage therapy	IV	[302]
12 natural lytic anti-*P. aeruginosa* bacteriophages (PP1131)	P	in vivo	Randomised phase ½ trial (27 patients with wound infections)	Reduced bacterial burden; minor adverse effects	Topical	[303]
PB AB08 PB AB25	A	in vivo	Mice infection model	35% survival rate	Intranasal	[304]
WCHABP1	A	in vivo	*Galleria mellonella* infection model	75% survival rate after phage administration		[283]
PD-6A3 and phage cocktail	A	in vivo	Sepsis mouse model	60% and 50% survival rate after phage therapy and phage cocktail	IP	[285]
Βϕ-R2096 sewage phage	A	in vivo	*Galleria mellonella* infection model	80% and 50% survival rate at 96 and 48 h.	Injection	[305]
	A	in vivo	Mouse model acute pneumonia	100%, 60% and 30% survival rate at day 12	Intranasal	
AB3P1, AB3P2, AB3P3, AB3P4, AB3P5	A	in vivo	Mice infection model	Bactericidal activity; 100% survival rate	IP	[306]
AB-PA01 lytic phages	P	in vivo	Case report (77-year old patient with adenocarcinoma)	Improved oxygenation; sedation ceased; bacterial eradication	IV Nebulisation	[307]

Ef, *Enterococcus faecium*; S, *Staphylococcus aureus*; K, *Klebsiella pneumoniae*; A, *Acinetobacter baumannii*; P, *Pseudomonas aeruginosa*; E, *Enterobacter* spp.
